# Tendo Sintomas de um Infarto Agudo do Miocárdio? Ligue para o seu Serviço Médico de Emergência Imediatamente!

**DOI:** 10.36660/abc.20220692

**Published:** 2022-11-01

**Authors:** Daniel Ferreira

**Affiliations:** 1 Hospital da Luz Digital Lisboa Portugal Hospital da Luz Digital, Lisboa – Portugal; 2 Hospital da Luz Lisboa Serviço de Medicina Intensiva Lisboa Portugal Serviço de Medicina Intensiva – Hospital da Luz Lisboa, Lisboa – Portugal

**Keywords:** Infarto do Miocárdio, Hospitalização, Mortalidade, Serviços Médicos de Emergência

A reperfusão oportuna das artérias coronárias ocluídas é fundamental para salvar as células miocárdicas isquêmicas em risco no infarto agudo do miocárdio com elevação do segmento ST (STEMI). Esse fato levou ao conceito de “Tempo é músculo” relacionado ao manejo do infarto agudo do miocárdio (IAM).^1^

Independentemente de qual estratégia de reperfusão é escolhida (lítica ou intervenção coronária percutânea (ICP), o tempo desde o início dos sintomas até a reperfusão bem-sucedida é fundamental para o prognóstico do paciente a curto e longo prazo.^2,3^

O tempo isquêmico total é o principal determinante do tamanho do infarto no STEMI. Ele foi dividido, desde o artigo de Terkelsen et al.,^4^ em ‘atraso do paciente’ e ‘atraso do sistema’, sugerindo que o último, mas não o primeiro, pode ser influenciado pelos sistemas e provedores de saúde.

O atraso do paciente (definido como o tempo entre o início dos sintomas e o primeiro contato médico (PCM), pode ser atribuído a várias características individuais, mas também societárias dos pacientes que apresentam STEMI e tem sido objeto de muitos estudos no passado. Depende em grande parte do conhecimento do paciente sobre os sintomas e a apresentação da doença coronariana.^5,6^

Por outro lado, as organizações hospitalares têm feito grandes esforços nas últimas décadas para alcançar a reperfusão oportuna das artérias coronárias ocluídas, reduzindo os tempos porta-agulha ou porta-balão e buscando modalidades melhores e mais seguras de terapias de reperfusão.

No entanto, há outra fração crucial do tempo total de isquemia sempre que o PCM ocorre no ambiente pré-hospitalar. O chamado atraso do Sistema Médico de Emergência (SME) pode ter um papel importante na redução do intervalo do primeiro contato médico (PCM) até a reperfusão, seja pelo início da terapia lítica na fase pré-hospitalar ou pelo redirecionamento dos pacientes para o hospital mais próximo com instalações de ICP disponíveis, ignorando assim os hospitais regionais que podem não ter essas instalações disponíveis.

As diretrizes STEMI da Sociedade Europeia de Cardiologia de 2017^7^ indicam que todos os componentes do atraso do sistema (incluindo o atraso do SME) representam a qualidade do atendimento, e recomenda-se mensurá-los como indicadores de qualidade.^7^

O atraso do SME depende em grande parte das políticas das autoridades locais, regionais ou nacionais quanto à organização dos serviços de ambulância e dos protocolos implementados no atendimento pré-hospitalar da síndrome coronariana aguda.

Neste número dos Arquivos Brasileiros de Cardiologia, Vieira et al.^8^ apresentam uma avaliação muito minuciosa da implantação de um sistema de ambulância pré-hospitalar sobre mortalidade por IAM no estado de Minas Gerais no sudeste brasileiro.^8^

Os principais desfechos deste estudo foram as taxas de mortalidade total e intra-hospitalar por IAM e as taxas de internação por IAM. Como mencionam os autores, esses desfechos foram escolhidos devido à sua alta relevância epidemiológica e clínica e ao maior potencial de associação com a implantação do sistema de ambulância.

Após um estudo muito abrangente dos múltiplos fatores que podem influenciar os desfechos do IAM durante o período 2008-2016, foi encontrada uma redução modesta (como assumido pelos autores), mas significativa na mortalidade atribuível ao IAM, e um dos principais fatores deste resultado poderia ser explicado pela implementação de um sistema de primeira resposta de ambulância pré-hospitalar.

Esses resultados estão de acordo com os relatados por Ferreira et al.,^9^ Eles publicaram uma análise da mortalidade por IAM nas diferentes regiões do Brasil, ao longo de 21 anos. Seus achados mostraram uma impressionante redução de 68% nas taxas de mortalidade por IAM na região Sudeste do Brasil, o que pode ser, pelo menos parcialmente, explicado pelos resultados do estado de Minas Gerais no período de sobreposição do estudo de Vieira et al.^8^

A boa articulação entre os serviços de emergência médica e os hospitais locais/regionais, com a adoção de protocolos comuns de atendimento ao IAM e a implantação de sistemas coronarianos acelerados, são bons exemplos de políticas de saúde e iniciativas de saúde pública que se traduzem em melhores resultados após um AMI.^10,11^

No entanto, nem mesmo o sistema de ambulância mais bem implementado ou os melhores protocolos de atendimento pré-hospitalar darão os resultados desejados se os pacientes não ativarem oportunamente a resposta do SME.

Grandes esforços devem ser feitos para educar os pacientes que apresentam sintomas que possam levantar a suspeita de ter um IAM para ligar imediatamente para o número de emergência do EMS e pedir assistência (que pode incluir o envio de uma ambulância com equipe e equipamentos adequados) em vez de ir aos serviços de saúde por meio de transporte próprio.
