# Supplementary material for: Tendo Sintomas de um Infarto Agudo do Miocárdio? Ligue para o seu Serviço Médico de Emergência Imediatamente!
Source: Arq Bras Cardiol. 2022 Nov 1;119(5):764–5. [Article in Portuguese] doi: 10.36660/abc.20220692 (PMC9750225; doi:10.36660/abc.20220692)
Supplement: Translation

# Translation: Having Symptoms of an Acute Myocardial Infarction? Call Your Emergency Medical Service Immediately!

**Authors:** Daniel Ferreira

**Affiliations:** 1 Lisboa Portugal Hospital da Luz Digital, Lisboa – Portugal; 2 Lisboa Portugal Serviço de Medicina Intensiva – Hospital da Luz Lisboa, Lisboa – Portugal

**Keywords:** Myocardial Infarction, Hospitalization, Mortality, Emergency Medical Services

Timely reperfusion of the occluded coronary arteries is critical to saving at-risk ischemic myocardial cells in acute ST-elevation myocardial infarction (STEMI). This fact led to the concept of “Time is Muscle” related to acute myocardial infarction (AMI) management.^1^

Independently of what reperfusion strategy is chosen (lytics or percutaneous coronary intervention (PCI)), the time from symptom onset to successful reperfusion is critical to the patient's short- and long-term prognosis.^2,3^

Total ischemic time is the major determinant of infarct size in STEMI. It has been divided, since Terkelsen et al.^4^ paper, into ‘patient delay’ and ‘system delay,’ suggesting that the latter, but not the former, can be influenced by the healthcare systems and providers.

The patient delay (defined as the time between the initiation of symptoms and the first medical contact (FMC), can be attributed to several individual, but also societal characteristics of the patients presenting with STEMI and has been the subject of many studies in the past. It largely depends on the patient's knowledge of coronary disease-related symptoms and presentation.^5,6^

On the other hand, hospital organizations have made large efforts in the last decades to achieve timely reperfusion of the occluded coronary arteries by reducing the door-to-needle or door-to-balloon times and looking for better and safer modalities of reperfusion therapies.

However, there is another crucial fraction of the total ischemic time whenever the FMC occurs in the pre-hospital setting. The so-called Emergency Medical System (EMS) delay can play a major role in the reduction of the interval from first medical contact (FMC) to reperfusion, either by starting lytic therapy in the pre-hospital phase or by redirecting the patients to the nearest hospital with available PCI facilities, thus bypassing the regional hospitals that might not have those facilities available.

The 2017 European Society of Cardiology STEMI guidelines^7^ indicate that all components of the system delay (including the EMS delay) represent the quality of care, and it is recommended to measure them as quality indicators.^7^

The EMS delay largely depends on the local, regional, or national authorities' policies regarding the organization of the ambulance services and the implemented protocols regarding acute coronary syndrome's pre-hospital care.

In this issue of *Arquivos Brasileiros de Cardiologia*, Vieira et al.^8^ present a very thorough evaluation of the implementation of a pre-hospital ambulance system on AMI mortality in the state of Minas Gerais in southeastern Brazil.^8^

This study's main outcomes were total and in-hospital mortality rates due to AMI and hospitalization rates AMI-related. As the authors mention, these outcomes were chosen due to their high epidemiological and clinical relevance and the highest association potential with the ambulance system implementation.

After a very comprehensive study of the multiple factors that might influence the AMI outcomes during the 2008-2016 period, a modest (as assumed by the authors) but significant reduction in mortality attributable to AMI was found, and one of the major factors of this outcome could be explained by the implementation of a pre-hospital ambulance first-response system.

These results are in line with the ones reported by Ferreira et al.^9^ They published an analysis of the mortality due to AMI in the different regions of Brazil over 21 years. Their findings have shown an impressive 68% reduction in AMI mortality rates in the Southeast region of Brazil, which can be, at least partially, explained by the Minas Gerais state results in the overlapping period of the Vieira et al.^8^ study.

The good coordination between the emergency medical services and the local/regional hospitals, with the adoption of common protocols regarding AMI care and the implementation of coronary fast-track systems, are good examples of health policies and public health initiatives that translate into better outcomes after an AMI.^10,11^

However, not even the better-implemented ambulance system or the best pre-hospital care protocols will give the desired outcomes if the patients do not timely activate the EMS response.

Large efforts should then be put into educating patients presenting with symptoms that might raise the suspicion of having an AMI to immediately call their EMS emergency number and ask for assistance (that might include the dispatch of an ambulance with the proper crew and equipment) rather than going to the health services by their own means of transportation.
